# Health Tract, No. 152

**Published:** 1865

**Authors:** 


### HEALTH TRACT, No. 152.

### DYSENTERY

Is literally a "difficulty among the intestines;" it is a discharge of blood from the bowels, accompanied with what has been aptly called "an atrocious pain." You feel as if you would be relieved by an evacuation, but when the attempt is made, there is a fruitless straining, termed *tenesmus*, and nothing comes of it, unless it be blood. The rectum, or last foot of the lower bowel, is the main seat of dysentery, which is commonly called "bloody flux." It should be always considered a dangerous disease. At first the discharges are odorless; but as the parts come more under the influence of the disease, they become disorganized, rotten, and insufferably offensive. Dysentery most abounds in hot, dry weather, and is oftenest caused by bad air, a sudden check of perspiration, or by whatever makes the skin of the body cold. In fact, dysentery may be considered an exaggerated or aggravated diarrhea—the latter is water, the former, blood. The great distinguishing features of dystentery are bloody passages, with a frequent, fruitless, and painful effort to stool. It is one of those diseases which are very apt to go on to a fatal termination, if let alone; a disease which is often made more speedily fatal by being ignorantly tampered with; and whether blood is passed from the bladder or the bowels, a skillful physician should be called in as promptly as possible, as promptly, indeed, as if it were an attack of cholera; but while he is coming, there are several things which may be safely done for the comfort of the sufferer, if not for his cure. The patient should not sit up a moment; should keep as quiet as possible; should eat absolutely nothing but boiled rice, or flour-porridge, and swallow bits of ice to the complete quenching of the thirst. A little cold flaxseed-tea may be swallowed from time to time. A favorite prescription of some of the old physicians of a past generation, and which is now said to be in vogue in Russia for several forms of diarrhea and dysentery is the use of raw meat—thus, take fresh beef, free from fat, scrape it into a pulp with a knife, season it with salt to make it more palatable, or with sugar for children, to whom begin with one teaspoonful three times a day, gradually increasing the amount as they become fond of it. Adults may use it by spreading it between two slices of stale bread. Its merit consists in its being easily digested, very nutritious, of small bulk, and readily assimilated to the system. It is well known that children having the summer complaint will ravenously eat, or rather chew or grind between their gums, a piece of the rind of bacon or ham, to which is attached half an inch of fat, and begin to improve in a few hours. The whites of forty eggs "whipped," and then sweetened with white sugar, and drank largely through the day, without any other food, is an admirable remedy in these ailments. Or for dysentery or protracted diarrhea take half a teacup of vinegar, with as much salt as it will take up, leaving a little excess of salt at the bottom, add boiling water until the cup is two thirds full, remove the scum, let it cool, and take one tablespoonful three times a day until relieved. It has not failed of cure in many hundred trials.

